# Proteins and Molecular Pathways Relevant for the Malignant Properties of Tumor-Initiating Pancreatic Cancer Cells

**DOI:** 10.3390/cells9061397

**Published:** 2020-06-03

**Authors:** Lisa Samonig, Andrea Loipetzberger, Constantin Blöchl, Marc Rurik, Oliver Kohlbacher, Fritz Aberger, Christian G. Huber

**Affiliations:** 1 Department of Biosciences, Bioanalytical Research Labs, University of Salzburg, A-5020 Salzburg, Austria; lisa.samonig@thermofisher.com (L.S.); constantin.bloechl@sbg.ac.at (C.B.); 2 Department of Biosciences, Cancer Cluster Salzburg, Molecular Cancer and Stem Cell Research, University of Salzburg, A-5020 Salzburg, Austria; andrea.loipetzberger@gmail.com; 3 Institute for Bioinformatics and Medical Informatics, University of Tübingen, Sand 14, 72076 Tübingen, Germany; rurik@informatik.uni-tuebingen.de (M.R.); kohlbacher@informatik.uni-tuebingen.de (O.K.); 4 Biomolecular Interactions, Max Planck Institute for Developmental Biology, Max-Planck-Ring 5, 72076 Tübingen, Germany; 5 Institute for Translational Bioinformatics, University Hospital Tübingen, Hoppe-Seyler-Str. 9, 72076 Tübingen, Germany; 6 Quantitative Biology Center, University of Tübingen, Auf der Morgenstelle 10, 72076 Tübingen, Germany; 7 Department of Biosciences, Cancer Cluster Salzburg, University of Salzburg, A-5020 Salzburg, Austria

**Keywords:** proteomics, cancer stem cells, CSCs, TICs, S100 proteins, S100A8, S100A9, LGALS3BP, MRP14, MRP8, MAC-2-BP, differential proteome analysis, functional assays, tumor grafting model

## Abstract

Cancer stem cells (CSCs), a small subset of the tumor bulk with highly malignant properties, are deemed responsible for tumor initiation, growth, metastasis, and relapse. In order to reveal molecular markers and determinants of their tumor-initiating properties, we enriched rare stem-like pancreatic tumor-initiating cells (TICs) by harnessing their clonogenic growth capacity in three-dimensional multicellular spheroid cultures. We compared pancreatic TICs isolated from three-dimensional tumor spheroid cultures with nontumor-initiating cells (non-TICs) enriched in planar cultures. Employing differential proteomics (PTX), we identified more than 400 proteins with significantly different expression in pancreatic TICs and the non-TIC population. By combining the unbiased PTX with mRNA expression analysis and literature-based predictions of pro-malignant functions, we nominated the two calcium-binding proteins S100A8 (MRP8) and S100A9 (MRP14) as well as galactin-3-binding protein LGALS3BP (MAC-2-BP) as putative determinants of pancreatic TICs. In silico pathway analysis followed by candidate-based RNA interference mediated loss-of-function analysis revealed a critical role of S100A8, S100A9, and LGALS3BP as molecular determinants of TIC proliferation, migration, and in vivo tumor growth. Our study highlights the power of combining unbiased proteomics with focused gene expression and functional analyses for the identification of novel key regulators of TICs, an approach that warrants further application to identify proteins and pathways amenable to drug targeting.

## 1. Introduction

Cancer represents a broad set of heterogeneous malignant diseases driven by distinct genetic and epigenetic alterations, where combinations of driver mutations and driver signals determine over time and space the malignant phenotype characterized by largely unrestricted proliferative capacity. Studies have shown that tumor initiation and growth are not stochastic events but processes controlled by a hierarchical organization within the heterogeneous tumor mass: malignant development and growth are fueled by a rare subpopulation of tumor-initiating cancer cells, frequently referred to as cancer stem cells (CSCs), with the ability of self-renewal and differentiation into the diverse cells that comprise the heterogeneous tumor bulk [1,2,3,4,5,6]. More recently, the concept of stem cell plasticity has been proposed for CSCs suggesting flexible reprogramming of CSCs into or from more differentiated phenotypes depending on the specific tumor niche [7].

CSCs can be highly metastatic and, due to a multitude of possible mechanisms, also therapy-resistant, e.g., their efficient self-detoxification machinery or quiescence, respectively, accounting for minimal residual disease and disease relapse [8]. Identifying the molecular players controlling the malignant properties of CSCs is therefore critical for the development of innovative multimodal treatments that include targeting of the tumor-initiating cancer cell population or its surrounding microenvironment [9]. In order to study molecular properties of cancer stemness, CSCs have been characterized in a multitude of cancer models and cancer cell lines employing transplantation, lineage tracing, or cell ablation assays [10,11,12]. As a result, clonal lines bearing stem cell-like properties could be described in numerous cancer entities by a specific set of markers, i.e., surface proteins, transcription factors, or miRNAs [13]. Although the size of the CSC population in a tumor or a specific cell line can vary significantly [14], the low abundance of CSCs in the majority of tumors makes their characterization using established high-throughput techniques challenging and frequently requires the application of error-prone amplification and/or selective enrichment before investigations at a molecular level can be performed.

Pancreatic ductal adenocarcinoma (PDAC) is one of the most aggressive forms of cancer, with a five-year survival rate of less than 5% [15]. This dreadful prognosis is generally a result of late diagnosis, highly aggressive metastatic behavior, and insufficient response to current treatments [16,17], including surgical resection, radiation-, and chemotherapy [18]. Moreover, only a low percentage of patients can be resected [19], and most therapies reduce tumor mass but fail to fully eliminate CSCs. There is thus high medical need for multimodal therapies specifically targeting pancreatic CSCs. Several populations of CSCs were recognized in pancreatic cancer. These populations are characterized by specific sets of markers (summarized in References [7,20]) such as CD44+/CD24+/ESA+ [21], CD133+ or CD133+/CXCR4+, the latter marking a metastatic population [11], high ALDH1 activity [22,23], c-Met [24], and more recently, a low activity of the 26S proteasome [25]. In contrast to the above marker proteins that were identified in CSCs of a variety of cancer entities, the PD2/Paf1 complex is a more specific CSC marker that was found to be upregulated exclusively in pancreatic and ovarian CSCs [26,27,28]. Novel drug candidates are currently evaluated that target critical CSC pathways such as c-Met or Hedgehog, Wnt, and Notch signaling responsible for CSC maintenance in many cancers (reviewed in References [9,29,30]). More specifically, pancreatic CSCs were efficiently targeted by pharmacological inhibition of the Alk4/7 receptor, which diminished their self-renewal capacity and made them susceptible to gemcitabine treatment [31].

In order to enlarge this knowledge and to identify novel drug targets, performing omics experiments is a powerful strategy for the generation of comprehensive data sets for the unbiased study of molecular and functional differences between selected tumor cell populations [32,33,34,35,36]. Despite the broad range of CSC markers, comparison of different CSC populations in the studies mentioned above showed only limited overlap between the populations. The lack of a universal CSC marker for cell enrichment as well as the heterogeneity and plasticity of CSCs has previously prompted us and others to establish undistorted methods for the enrichment of tumor-initiating pancreatic cancer cells solely based on phenotypic growth properties (i.e., clonal growth in three-dimensional tumor spheroid cultures) rather than ambiguous surface marker expression [37]. Specifically, high clonogenic growth and sphere formation in semi-solid three-dimensional (3D) cultures represent efficient selection criteria for the enrichment of tumor-initiating cells (TICs) with pronounced self-renewal capacity similar to CSCs [37,38,39]. Notably, employing this selection process allows expansion of TICs to levels sufficient for performing proteome analysis without any amplification or sorting steps that may distort the original ratio of biomolecules within the stem cell-like TIC fraction. 

Previous reports conducted proteomics experiments to characterize CSCs both from cell lines as well as xenograft models yet employing fluorescence-activated cell sorting staining CD44 and CD24 to enrich CSCs [40,41]. Unique regulations of the cell cycle, proliferation, apoptosis, and cellular differentiation were reported. More recently, comparative proteomics (PTX) of pancreatic cancer stem cells revealed aberrant metabolic pathways, thereby identifying possible drug targets, such as the fatty acid pathway inhibited by cerulenin and as well as the mevalonate pathway inhibited by atorvastatin [42].

In this study, we employ an unbiased PTX approach to reveal in detail the molecular complexity of rare TICs of human pancreatic cancer enriched in 3D spheroid cultures in comparison to nontumor-initiating cells grown in a 2D approach utilizing well-characterized cell line models. This global approach aims at the discovery of unique proteins and biochemical pathways enriched in pancreatic TICs and, in combination with hypothesis-driven approaches, shall lead to the identification of novel key genes with a critical role in the determination of the malignant phenotype of pancreatic CSCs. Following this unbiased and hypothesis-driven combined approach, we identified S100A8, S100A9, and LGALS3BP as proteins overexpressed in the tumor-initiating cell populations. Functional assays revealed a critical contribution of these gene products to the malignant behavior of pancreatic cancer cells in vitro and in vivo. These findings increase our current understanding of the molecular determinants of tumor-initiating cells and may therefore contribute to the development of innovative therapies based on targeting of TIC- and CSC-specific factors in pancreatic cancer.

## 2. Materials and Methods

### 2.1. Cell Culture

L3.6sl and L3.6pl pancreatic cancer cells were kindly provided by Dr. J. Fidler [43]; Panc1 cells were purchased from the American Type Culture Collection (ATCC No. CRL-1469). For 2D (2-dimensional) cell culture, L3.6sl, L3.6pl, and Panc1 cells were grown in Dulbecco´s Modified Eagle Medium (DMEM) (PAA Laboratories GmbH, Pasching, Austria) supplemented with 10% fetal bovine serum (FBS) (PAA Laboratories GmbH), penicillin (62.5 µg × mL^−1^, Invitrogen, Carlsbad, CA, USA), and streptomycin (100 mg × mL^−1^, Invitrogen) at 37 °C in a humidified atmosphere containing 5.0% CO_2_. For 3D cell culture, L3.6sl, L3.6pl, and Panc1 cells were seeded at clonal density in 12-well plates in 800 µL 0.40% Select Agar on top of 800 µL 0.50% Select Agar (Life Technologies, Vienna, Austria), according to standard protocols, to prevent cell adherence. The agar layers were covered with 400 µL culture medium. For L3.6sl and L3.6pl, 1 × 10^4^ cells and, for Panc1, 5 × 10^3^ cells were seeded and grown for 6 weeks at 37 °C in a humidified atmosphere containing 5.0% CO_2_. For details of TIC enrichment and characterization (tumor-initiating capacity, stemness gene expression signatures, and self-renewal), see Reference [37]. Sphere growth was documented on a stereomicroscope with NIS image capture system and quantified using Colony Counter (Microtech Nition, Funabashi City, Japan).

### 2.2. Preparation of Protein Extracts, Digestion, and Labeling

Two replicates of Dulbecco’s phosphate buffered saline (PBS, 1×, without calcium and magnesium, PAA laboratory GmbH)-washed 2D and 3D cells were suspended in PBS containing a complete ethylenediaminetetraacetic acid (EDTA)-free protease inhibitor cocktail (Roche Diagnostics, Penzberg, Germany). Single clonal spheroids enriched for pancreatic TICs were harvested by aspiration and were combined for lysis to reach a number of cells amenable for proteomics. Upon addition of 1:1 (*w*/*w*) trifluoroethanol (Fluka Analytical, Buchs, Schweiz), whole cell lysates were prepared by ultrasonic solubilization in a Bioruptor (Diagenode Inc, New Jersey, NJ, USA) followed by addition of 50 mmol × L^−1^ tris-(2-carboxyethyl)phosphine (Sigma Aldrich Chemie, Steinheim, Germany) for reduction at 60 °C for one hour in a thermomixer (Eppendorf, Hamburg, Germany). The alkylation of cysteine residues was carried out in 200 mmol × L^−1^ iodoacetamide (Bioultra, Sigma Aldrich) at room temperature for 30 min protected from light. Acetone (Chromasolv plus, for HPLC, ≥99.9%, Sigma Aldrich) precipitation was performed over night at −20 °C to isolate the proteins. The samples were centrifuged at 4.0 °C and 14,000 rpm for 5.0 min (Hermle Labortechnik GmbH, Wehringen, Germany). After discarding the supernatant, the air-dried pellets were dissolved in 100 µL 0.50 mol × L^−1^ triethylammonium bicarbonate (Sigma Aldrich). Before digestion, the amount of protein in the extracts was determined via a Bradford assay (Sigma Aldrich) assay using an Infinite 200Pro (Tecan Group Ltd., Männedorf, Switzerland) instrument. The samples were digested with Porcine Sequencing Grade Modified Trypsin (Promega, Madison, WI, USA) in a protein:enzyme ratio of 100:1 at 37 °C overnight.

From each sample, a 50-µL sample solution volume containing 100 µg of peptides was used for labeling. The isobaric tags for relative and absolute quantitation (iTRAQ^®^) kit was obtained from Sciex (Framingham, MA, USA). The required volume of peptide solution was evaporated to dryness in a vacuum concentrator (Concentrator Plus, Eppendorf, Hamburg, Germany) and redissolved in 20 µL iTRAQ dissolution buffer. The iTRAQ reagents were brought to room temperature and dissolved in 70 µL ethanol (Sigma Aldrich), and 70 µL dissolved labeling reagent was added to every sample. The peptide-reagent mixture was incubated for 1 h at room temperature. After quenching of unreacted label, the samples were completely dried in a vacuum concentrator and redissolved in 0.050% aqueous trifluoroacetic acid (TFA) to a final concentration of 1.0 µg/µL. For quantitative comparison of the two cell lines L3.6sl and L3.6pl grown under 2D or 3D conditions, the iTRAQ channels of the isobaric labels were assigned to the corresponding experiments as follows: 114: L3.6sl 2D; 115: L3.6pl 2D; 116: L3.6sl 3D; and 117: L3.6pl 3D. Equal amounts of labeled peptides from each lysis were pooled and subsequently analyzed in a 4-plex iTRAQ HPLC-MS/MS run measuring two technical replicates.

### 2.3. High-Performance Liquid Chromatography and Mass Spectrometry

Five micrograms of peptides was separated in an ion-pair reversed-phase high-performance liquid chromatography (IP-RP-HPLC) system (Model UltiMate U3000, Thermo Scientific, Germering, Germany) controlled by a computer-based data system (Chromeleon^®^ Chromatography Data System, version 7.1.0.898, Thermo Scientific, Germering, Germany) using a monolithic poly-(styrene-divinylbenzene) separation column (150 mm × 0.20 mm i. d., produced in house [44]). The separations were performed at 45 °C at a flow rate of 1.0 µL × min^−1^ and a linear gradient of 0%–40% acetonitrile (Chromasolv Gradient grade, for HPLC, 99.9%, Riedel de Haen, Steinheim, Germany) in 0.050% aqueous TFA in 300 min.

The HPLC system was hyphenated to a linear ion trap-Orbitrap mass spectrometer (Model LTQ Orbitrap XL, Thermo Scientific, Bremen, Germany) via an electrospray ionization source (Thermo Scientific) and a PicoTip emitter (10 µm I.D., New Objective Inc., Woburn, WA, USA). Three measurements per sample were performed in positive ionization mode, a heated capillary temperature of 250 °C, and the voltage set to 1.4 kV. During the data acquisition of 300 min, MS^1^ FTMS full scans were acquired in the Orbitrap from 450–2000 *m*/*z* at a resolution of 60,000 with an automatic gain control (AGC) setting of 10^6^ and a maximum ion injection time of 100 ms. Peptides were identified in parallel in the linear ion trap consecutively using the three most intense precursor ions for fragmentation by collision-induced dissociation (CID) with a normalized collision energy of 35.0 using an isolation window of 2 Da, AGC 10.000 with 100 ms maximum injection time, and a dynamic exclusion window +/−10 ppm of 30 s duration. The reporter ions were detected upon higher-energy collision-induced dissociation (HCD) with a normalized collision energy of 40.0 also using a top 3 method at a resolution of 7500 with an isolation width of 4.00 Da and an activation time of 40.0 ms. Singly charged ions were excluded. To gain more identifications, identified peptides were rejected from data-dependent scans in the following run by the use of exclusion lists [45]. Precursor masses of peptide candidates identified in the previous run were set on the instrument’s global exclusion list within a retention time window of ±42 min. For each set of 4-plexed samples, an individual exclusion list was generated. Mass spectrometric data was analyzed with Xcalibur^®^ software, version 2.0.7 SP1 (Thermo Fisher Scientific Inc.). The proteomics data has been deposited to the ProteomeXchange Consortium via the PRIDE partner repository [46] with the dataset identifier PXD018585.

### 2.4. Data Analysis

For identification of peptides and corresponding proteins, CID and HCD spectra were converted into the open file formats mzML and mzXML with the software tool MSConvert (ProteoWizard Tool, Version 1.5.2, http://proteowizard.source-forge.net/) and the open-source software library OpenMS (Version 2.5, http://open-ms.sourceforge.net/) [47,48]. MaxQuant (version 1.6.12.0.) was used for peptide and protein identification applying a 5% false-discovery rate (FDR) for both, peptide-spectrum matches (PSMs) and protein identifications [49]. Databases searches were performed against the human swiss prot database containing 20,365 entries (access: 30.03.2020) [50]. Relative quantification was based on the iTRAQ isotope-labeled reporter ions. Basic filtering steps including removal of decoy hits, peptides identified by site, identified potential contaminants, and proteins missing quantitative data were performed using the Perseus software (version 1.6.12.0) [51]. Quantitative data was log_2_-transformed and subsequently normalized by subtraction of the median. Protein identification data was further processed and visualized with the statistical program R (version 3.6.1), SIMCA 13.0.3, and GraphPad Prism 8.0.2. For principal component analysis (PCA), protein abundances were preprocessed by batch-correction for the two iTRAQ replicates using SVA analysis [52], centered and transformed to unit variances. Statistical significance of differences in protein regulations was tested using a paired t-test correcting for multiple comparisons applying Benjamini–Hochberg (FDR 10%). Statistically significantly regulated proteins were further analyzed using Ingenuity Pathway Analysis (IPA, Ingenuity^®^ Systems, Qiagen, Venlo, Netherlands).

### 2.5. RNA Isolation and qPCR

For RNA isolation, cells grown in 2D cultures (non-TICs) were lysed in total RNA isolation-reagent (Molecular Research Center Inc., Cincinnati, OH, USA) and isolated according to the manufacturer’s protocols, followed by LiCl purification. Spheres grown in 3D (enriched for TICs [37]) were isolated by aspiration, washed three times with PBS, lysed in TRI, and processed as described above. cDNA was synthesized using M-MLV reverse transcriptase RNase H Minus, Point Mutant (Promega). qPCR was performed on a Rotor Gene Q (Qiagen, Hilden, Germany) using GoTaq qPCR Master Mix (Promega, Fitchburg, WI, USA). For primer sequences, see Table 1.

### 2.6. shRNA and Migration Assay

shRNA constructs were purchased from the Sigma-Aldrich mission TRC library (St. Luis, MO, USA). Construct IDs are listed in Table 2. Lentivirus production and transduction were performed as previously described [53]. Transduced cells were selected for puromycin resistance and knockdown efficiency validated by qPCR.

shRNA transduced pancreatic cancer cells were seeded in culture inserts (ibidi, Martinsried, Germany) forming a silicone chamber with two wells, providing a 500-µm gap between the two cell growth areas. Cells were grown to 100% confluence and starved for 12 h with 0.50% FBS to prevent proliferation during the migration experiment. Removal of the culture insert introduces a 500-µm gap between cells, and the ability and speed of the perturbed cells to migrate into the gap and overgrow the cell free area was used as readout. Cell movement was assessed after 0.0, 6.0, 12.0, and 24.0 h and documented using a transmission light microscope with ProgRes capture system (Jenoptik GmbH, Jena, Germany).

### 2.7. Mouse Models

For xenograft studies, 1 × 10^5^ L3.6sl cells suspended in 25% Matrigel (BD Laboratories, Franklin Lakes, NJ, USA) were injected subcutaneously into the flanks of CD1-Foxn1^nu/nu^ nude mice (Charles River Laboratories, Wilmington, MA, USA); 1 × 10^5^ L3.6sl cells stably expressing either non-targeting control shRNA or the respective S100A8 and S100A9 shRNA were grown under standard 2D culture conditions (see Section 2.1.) and injected into the left and right lower flanks of nude mice, respectively. Tumor growth was monitored in five individuals for each shRNA construct over the next weeks until the tumors reached a maximum volume of 500 mm^3^. Nude mice were inoculated at the age of eight weeks. Effect-size-based calculation of mouse numbers to reach statistical significance was done using G*power software [54,55]. Tumor dimensions were measured twice a week, and tumor volume V was calculated as V = 1/6 × π × l × w × h, where l, w, and h are the length, width, and height of the tumor, respectively. All animal experiments were done in the animal facility at the University of Salzburg according to institutional and federal guidelines (animal handling application number GZ 66.012/2-BrGT/2007).

## 3. Results

### 3.1. Experimental Strategy for Proteomics and Functional Analysis

**Figure 1:**
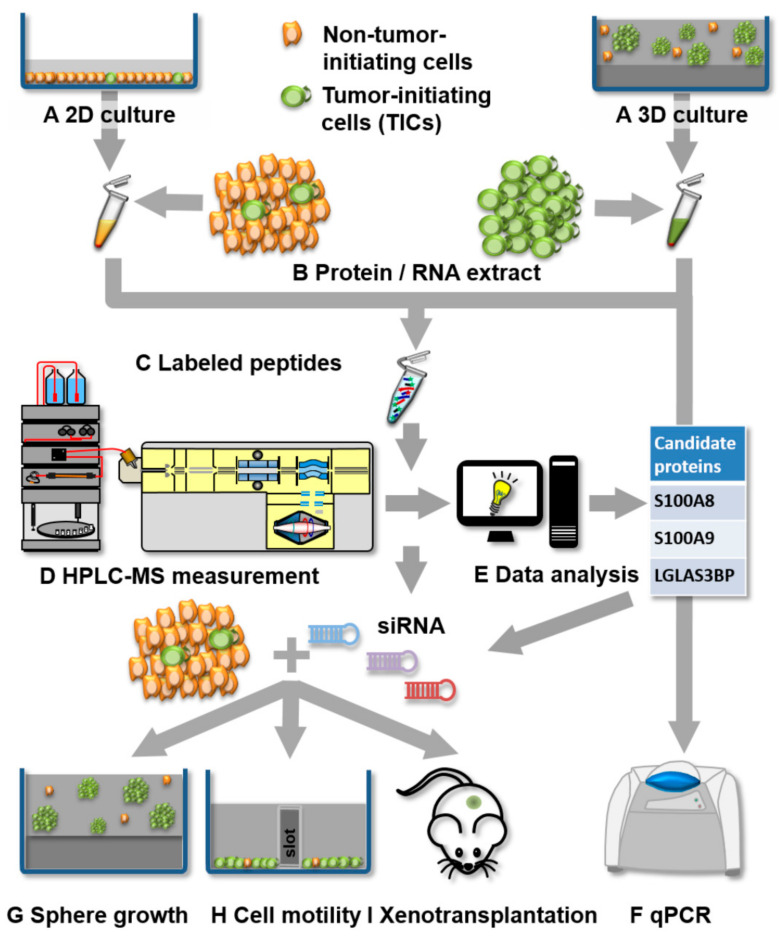
Workflow of differential quantitative proteomics of pancreatic cancer stem cells (CSCs) and non-CSCs followed by gene expression analysis and biological validation of selected target genes: Sample preparation involves cell culture (**A**), protein/RNA extraction (**B**), and protein digestion and labeling (**C**). Measurement of differential protein expression is performed by HPLC-MS/MS (**D**) and statistical analysis (**E**): Differentially expressed candidate proteins are verified at the transcript level by qPCR (**F**). Finally, candidate proteins/genes are validated using functional assays such a sphere growth (**G**), cell motility (**H**), or tumor initiation and growth in xenotransplants (**I**) upon siRNA-mediated knockdown.

In order to identify candidate proteins involved in the determination of the malignant phenotype of pancreatic TICs, we applied an unbiased differential proteomics approach followed by candidate-based gene-expression analysis and functional studies. An overview of the experimental setup, data evaluation, and target validation strategy is illustrated in Figure 1. Two well-established and characterized human metastatic pancreatic adenocarcinoma cancer cell lines, L3.6sl and L3.6pl. [43], served as cellular in vitro models for studying pancreatic TICs. We employed our established protocol for TIC enrichment and expansion [37] to compare TIC-enriched and non-TIC populations by culturing both cell lines under standard 2D planar conditions, mimicking the tumor bulk and, under 3D conditions, enriching TICs in multicellular spheres (Figure 1A). Proteins as well as RNA were extracted from both cellular samples (Figure 1B). In addition, tumor-initiating capacities of cells derived from 3D spheres in comparison to cells grown in 2D conditions were verified by limiting dilution experiments in a xenograft assay (Supplementary Figure S1). 

A tryptic protein digest of each cell line from 2D and 3D cultures was subjected to iTRAQ labeling (Figure 1C), subsequently combined, and characterized by HPLC-MS/MS for differential proteome analysis (Figure 1D). After statistical processing, these unbiased hits were evaluated by a systematic literature-based screen to identify putative candidate proteins contributing to the phenotype of pancreatic TICs (Figure 1E). Further, Ingenuity Pathway Analysis (IPA) was used to evaluate the data sets and to identify pathways involved in cancer stemness and tumorigenesis. Overexpression of significantly regulated candidate genes was corroborated through targeted, quantitative PCR (Figure 1F). Finally, to evaluate the contribution of chosen targets to the oncogenic TIC phenotype, we perturbed TIC-enriched genes by RNA interference and consequently monitored the effect of gene inhibition in functional assays including clonogenic sphere growth in 3D (Figure 1G), cell migration (Figure 1H), and in vivo tumorigenesis assays (Figure 1I).

### 3.2. Differential Proteome, Gene Expression, and Pathway Analysis

**Figure 2:**
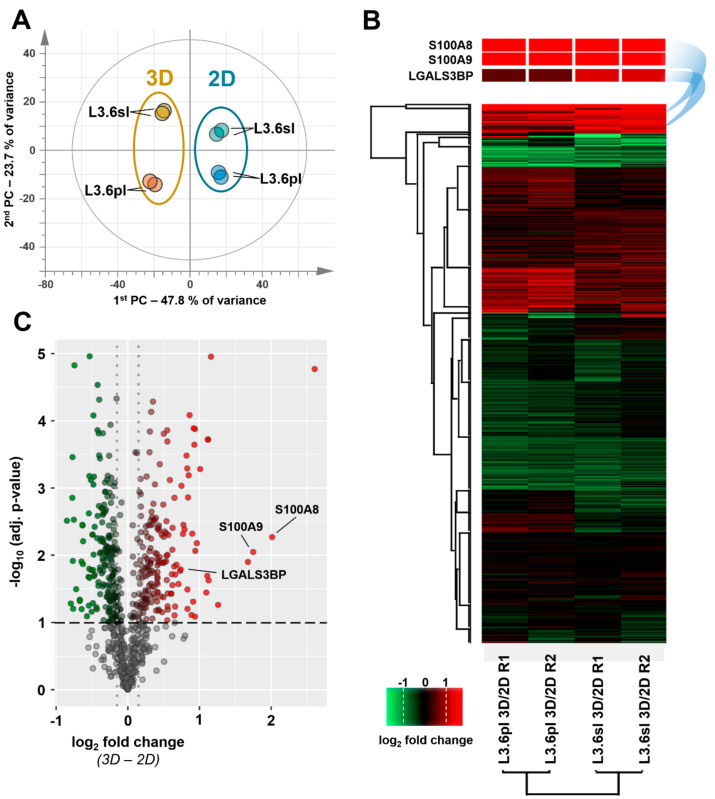
Principal component analysis (PCA) of the combined PTX dataset (**A**): Clusters of tumor-initiating cells (TICs) (3D) and non-TICs (2D) are indicated in orange and blue, respectively. Clustered heat map of log_2_-fold changes between TICs and non-TICs in L3.6sl and L3.6pl samples (green = downregulation, red = upregulation) (**B**). Proteins and samples, respectively, were grouped by means of hierarchical clustering. The top three rows show a magnified excerpt demonstrating the differences in expression of S100A8, S100A9, and LGALS3BP. Volcano plot depicting log_2_-fold changes (3D vs 2D) plotted against the negative decadic logarithm of the adjusted p-value (**C**): Adjusted p-values were assessed by a paired t-test correcting for multiple comparison with Benjamini–Hochberg at an FDR of 10%. Four replicates each were considered for 3D and 2D cultured cells. Nonsignificantly altered proteins are situated below the horizontal dashed line (grey dots). Proteins being significantly regulated are marked by gradually increasing tinting strength of either green (downregulation) or red (upregulation).

Multiplexed samples obtained from two independent biological replicates of both pancreatic cancer cell lines, L3.6sl and L3.6pl, in both cell culture conditions (2D vs 3D) were prepared and analyzed; 729 proteins were identified and quantified throughout all eight samples, while 828 and 851 proteins, respectively, were quantified in one of the two iTRAQ experiments. Detailed qualitative and quantitative data on PTX experiments are provided as supplement (SI-Excel). Principal component analysis revealed a strong clustering of TICs and non-TICs, explaining nearly 50% of the variance in the dataset (Figure 2A).

This strong overlap of the two cell lines prompted us to use quantitative PTX data in a combined manner to screen for reliable genes underlying TIC properties. The second grouping observed was governed by the type of cell line accounting for about 24% of the variance. In addition, biological replicates showed a clear overlay in the PCA. A global view on the dataset is provided in form of a clustered heat map (Figure 2B). Quantitative changes between TICs and non-TICs are represented in a color code for all four pairs of samples. Furthermore, the PTX dataset was screened for significantly regulated proteins by means of statistical testing. A graphic summary is depicted in the form of a volcano plot (Figure 2C). A total of 410 proteins was found to be significantly regulated, whereas 195 of them were up- and 215 downregulated in TICs compared to the non-TICs. For manual candidate discovery, an arbitrary cutoff ratio of ±1.3-fold change of the relative protein expression was set. This threshold for relative changes in protein expression was considered appropriate for filtering suitable preliminary candidate proteins. In addition to this unbiased data-driven strategy, we applied a more hypothesis-driven approach to identify proteins with a functional role in the determination of the malignant phenotype of pancreatic TICs. Therefore, a systematic literature search and additional analyses based on the data from The Cancer Genome Atlas (TCGA) for expression and poor survival were performed (data not shown), leading to the nomination of functionally relevant candidates. This approach finally yielded a candidate list of eight differentially regulated proteins extracted from the PTX experiments (Table 3) that were subjected to further validation via gene expression analysis.

Upregulation at transcript level in tumor-initiating cells was verified by means of qPCR. Table 3 collects representative results of L3.6sl cells for eight different genes with highly significant regulation at the protein level.

Based on PTX, qPCR, and literature data, three genes with a putative function in pancreatic TICs were nominated: the calcium-binding proteins S100A8 and S100A9 and the lectin, galactoside-binding, soluble 3-binding protein LGALS3BP (labeled in Figure 2B,C). To provide additional verification, two unique peptides were annotated for each candidate, confirming their unambiguous identification by PTX (Supplementary Figure S2).

**Figure 3:**
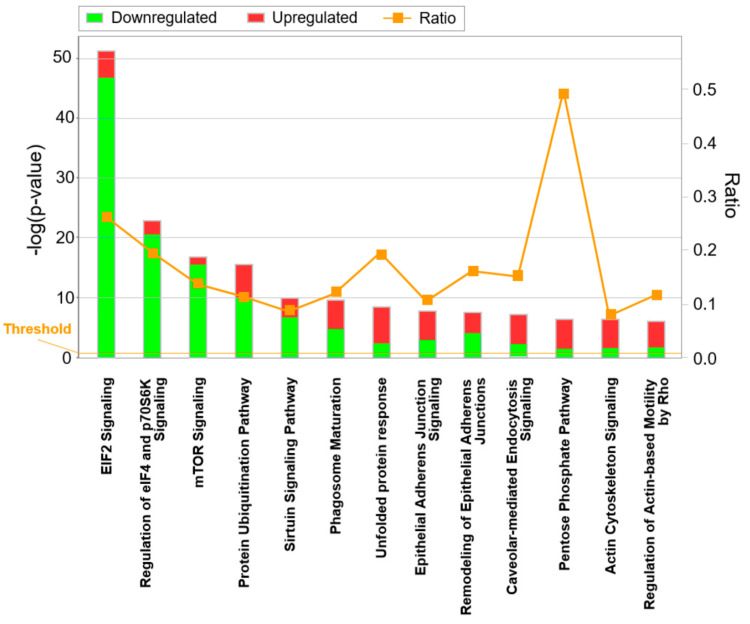
Canonical Ingenuity Pathway Analysis (IPA) pathways altered in pancreatic TICs ranked by significance: For each pathway (x-axis), ratios of significantly up- or downregulated proteins are represented in red and green, respectively. −log(*p*-value) is the negative decadic logarithm of the probability that the observed association between a specific pathway, and the dataset is only due to random chance according to Fisher’s exact test; the ratio is the quotient between the number of genes detected in our particular analysis (identified via the corresponding proteins) and the number of genes contained in the Ingenuity database for the specific canonical pathway.

In addition to candidate-based screening of the acquired PTX data, a more comprehensive evaluation of all significantly changed proteins was carried out using pathway analysis software. Figure 3 shows significantly altered canonical pathways identified by IPA ranked by their p-value. These pathway analyses demonstrate a prominent downregulation of translational activity in TICs paired with modulated migratory capacities. In addition, a more comprehensive figure using a −log(*p*-value) cutoff of 5 (Supplementary Figure S3) shows additional pathways and reveals a pronounced upregulation of glycolysis. Furthermore, the interplay of these regulated pathways was examined in detail (Supplementary Figure S4). Intriguingly, a number of altered pathways controlling migratory capacities, translation, and energy metabolism was interconnected. Finally, our pathway suggests key involvement of S100A8 and S100A9 in a putative signaling network (Supplementary Figure S5).

### 3.3. Candidate Verification by Functional Assays and Xenograft Analysis

**Figure 4:**
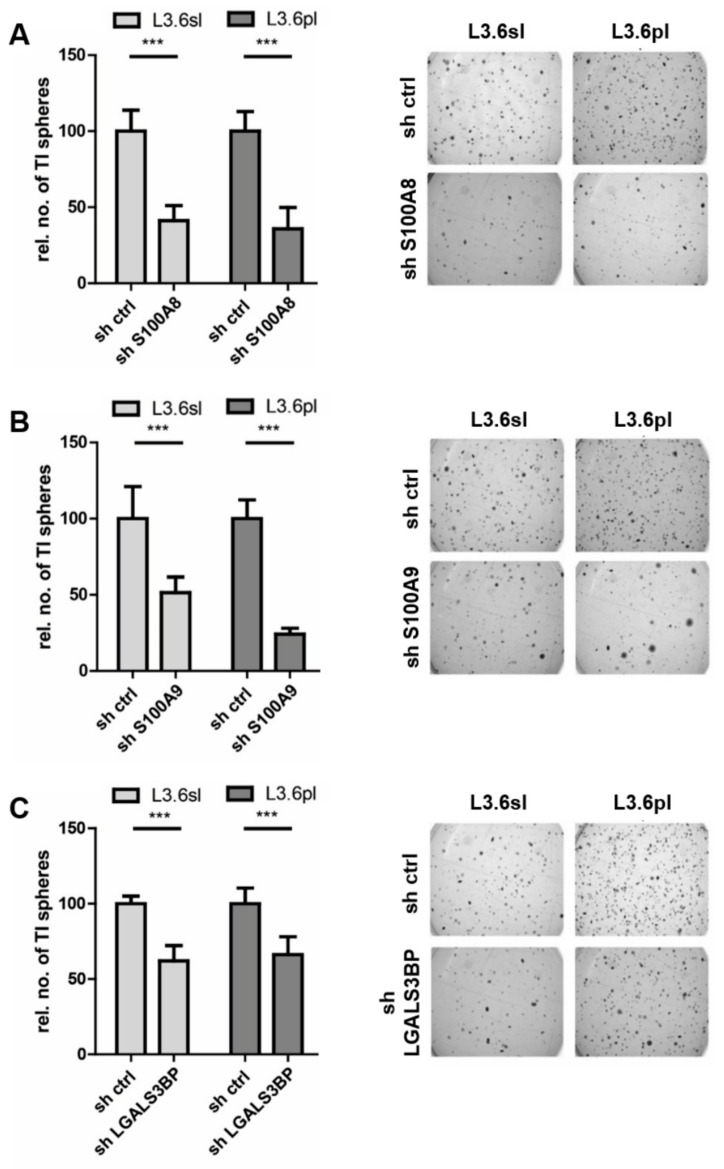
Knockdown of TIC genes S100A8, S100A9 and LGALS3BP reduces tumor-initiating (TI) sphere formation of pancreatic TICs. Relative numbers (rel. no.) of spheres upon transduction with control shRNA (sh ctrl) or shRNA against S100A8 (**A**), S100A9 (**B**), and LGALS3BP (**C**) are given as bar charts (left column). Representative images of TI sphere growth are depicted on the right column. Error bars represent standard error of the mean. Statistical significances were assessed by an unpaired t-test (* for *p* ≤ 0.05, ** for *p* ≤ 0.01, and *** for *p* ≤ 0.001, *n* = 12). Additional validation experiments were conducted in Panc1 cells (Supplementary Figure S7).

To corroborate the biological function of the three candidate TIC genes/proteins, we performed RNAi perturbation experiments by lentiviral shRNA knockdown of S100A8, S100A9, and LGALS3BP in the human pancreatic cancer cell lines L3.6sl and L3.6pl and, for validation, also in KRAS-mutated Panc1 cells. In all knockdown experiments, a non-target shRNA served as control. The efficiency of the individual shRNA constructs was verified by qPCR (Supplementary Figure S6). Figure 4 and Supplementary Figure S7 show that individual knockdowns of S100A8, S100A9, and LGALS3BP significantly reduced the formation of tumor-initiating (TI) spheres in all three pancreatic cancer cell lines, i.e., L3.6sl (Figure 4), L3.6pl (Figure 4), and Panc1 (Supplementary Figure S7). Inhibition of S100A8 had the most pronounced effect on tumor-initiating sphere formation, reducing the number of spheres by more than 50 percent in all cell lines studied. 

**Figure 5:**
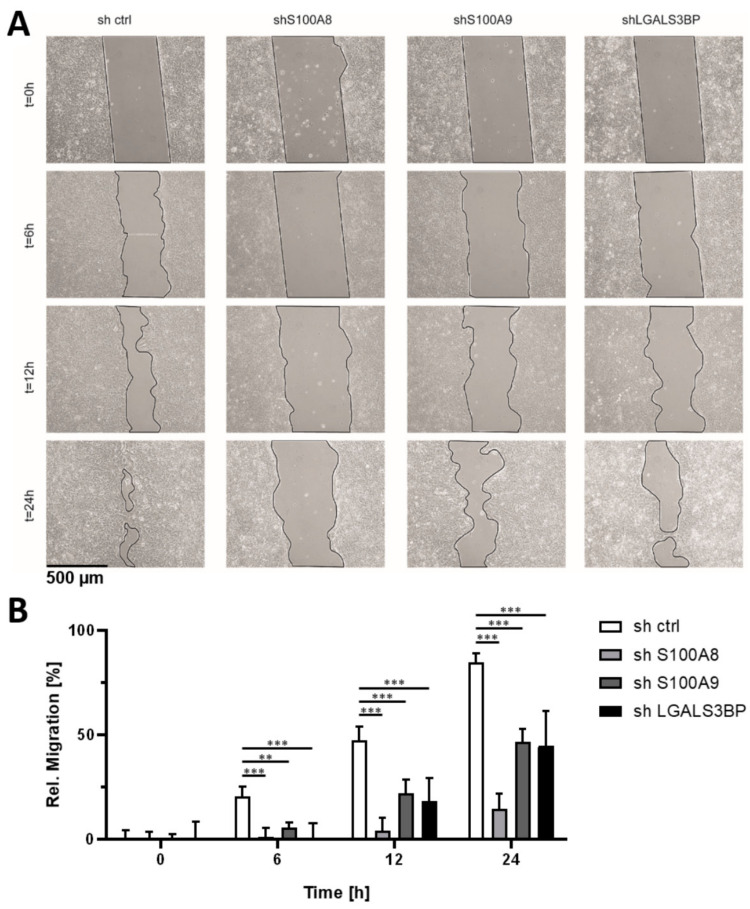
Inhibition of S100A8, S100A9, and LGALS3BP expression impairs migration of pancreatic cancer cell line L3.6sl. (**A**) Representative images of the specific knockdown cells (shS100A8, shS100A9, and shLGALS3BP) compared to non-target control shRNA cells (sh ctrl) shown are after 0.0, 6.0, 12.0, and 24.0 h. Black lines enclose areas free of migratory cancer cells. Quantification of migration ability over time (**B**): 0.0 h control shRNA area was set to 0%; all other values were normalized accordingly. Measurements were done with the ProgRes Software. Error bars represent standard deviations of six measurements. Statistical significances were assessed by a two-way ANOVA followed by a Dunnett’s post-hoc test (* for *p* ≤ 0.05, ** for *p* ≤ 0.01. and *** for *p* ≤ 0.001, *n* = 6).

Next, we analyzed the effect of TIC gene knockdown on the migratory ability of human pancreatic cancer cells as another readout for their tumorigenicity. For this purpose, we applied a modified scratch assay performed under starving conditions to minimize the effect of cell proliferation (cell proliferation was found to be negligible during the observation period; data not shown). As revealed in Figure 5, shRNA-mediated knockdown of S100A8, S100A9, and LGALS3BP resulted in a strong inhibition of cell migration as evidenced by the reduced ability to close the “scratch” gaps when compared to control cells.

**Figure 6:**
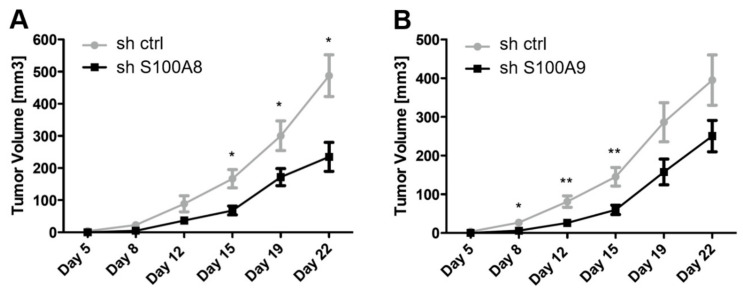
Inhibition of S100A8 and S100A9 in pancreatic cancer cell line L3.6sl reduces in vivo tumor growth. L3.6sl pancreatic cancer cells transduced with non-target control shRNA (sh ctrl) or with sh S100A8 (**A**) or sh S100A9 (**B**) were injected subcutaneously into immune-incompetent mice, and tumor growth was monitored over a period of 22 days. Error bars represent standard error of the mean. Statistical significances were assessed by a two-way ANOVA followed by a Dunnett’s post hoc test (* for *p* ≤ 0.05, ** for *p* ≤ 0.01, and *** for *p* ≤ 0.001, *n* = 5).

Finally, as knockdown of S100A8 and S100A9 revealed the most prominent effect on clonogenic growth and cell migration, we tested the in vivo relevance of our findings in tumor xenograft assays. We transplanted L3.6sl cells stably transduced with lentiviral shRNA either targeting S100A8 or S100A9 (xenografting was conducted as depicted in Supplementary Figure S1) onto the flank of athymic mice by subcutaneous injection and monitored tumor growth over a period of 22 days (Figure 6). Notably, knockdown of S100A8 and S100A9 significantly reduced tumor growth in nude mice. The delay in tumor engraftment due to perturbation of S100A8 and S100A9 further corroborates our experimental findings and suggests a prominent role of these two proteins regarding cancer stemness in pancreatic TICs.

## 4. Discussion

In the present study, we employed an unbiased enrichment method for pancreatic TICs followed by a quantitative proteomics approach comparing TICs to non-TICs in order to identify specifically enriched proteins. We provide proof of concept that coupling the unbiased PTX to candidate-based functional studies is an efficient strategy to identify TIC enriched proteins with a key role as determinants of the malignant characteristics of pancreatic TICs. Such strategies have been employed but are still urgently needed for the development of novel CSC/TIC inhibitor compounds to be included in current multimodal therapies mainly targeting the tumor bulk. In pancreatic ductal adenocarcinoma and possibly other malignant entities, CSC-targeted therapeutic interventions may have the chance to tackle this life-threatening disease and to improve its dismal prognosis [9]. In addition, PTX approaches targeting the secretome of cancer cells have been proven a valuable tool for biomarker discovery in pancreatic cancer [56,57].

One major limiting factor for most omics approaches is the low abundance of CSCs [41]. We have overcome these limitations on the one hand by enriching CSC-like TICs in spheroid cultures as previously demonstrated [37] and on the other hand by multiplexing and pooling of our samples. A global PTX data analysis employing PCA and a clustered heat map revealed a specifically altered proteome after enrichment for TICs in spheroids in both cell lines (Figure 2). As anticipated, additional grouping due to differences in the two cell lines was observed. However, a coherent overlay of the biological replicates suggests a consistent proteomic composition and assures cell culture conditions and experimental design being sufficiently stable and reproducible. In summary, 410 proteins were found to be significantly altered between TICs and non-TICs additionally highlighting a drastically altered TIC proteome. Finally, a detailed proteomics data analysis step corroborated by qPCR and literature search enabled the nomination of three candidate proteins. 

In a previous study, Dai et al. [41] employed flow cytometry with different surface markers to enrich pancreatic CSCs and bulk tumor cells from xenograft tumors. The two populations were compared by differential proteome analysis using capillary isoelectric focusing-nano HPLC-MS/MS. Among 1159 identified proteins, 169 were found to be differentially expressed. The overlap of regulated proteins between Dai et al. and our study is moderate, specifically 5 of 39 upregulated proteins (12.8%) and 19 of 130 downregulated proteins (14.8%) were also significantly altered in our study (Supplementary Figure S8 and SI-Excel). We believe that these differences are due to different cell isolation and enrichment procedures and to the substantial heterogeneity between patient samples.

However, when comparing the proteomics study of CD24+ pancreatic cancer cells enriched from patient material [58] with our data generated from two human pancreatic cancer cell lines, there is a clear match at the level of pathways differentially regulated in CSCs and tumor bulk cells. Like Zhu and coworkers [58], we found regulation of EIF2, EIF4, p70S6K, and mTOR signaling as the primary pathways differentially active in the CSCs/TICs and bulk/non-TIC population (Figure 3 and Figure S3). This agreement of our findings with data obtained with patient samples supports the relevance of our findings to human pathology.

The top canonical pathways affected in our pancreatic TIC populations, as revealed by IPA (Figure 3, Figures S3 and S4), included key metabolic and translational pathways that may be highly relevant for proliferation and energy supply of CSCs. Therefore, not only pathways like Hedgehog, Wnt, and Notch may represent interesting targets for therapeutic interventions [9] but also metabolic processes could be prospective candidates [59,60]. Indeed, recently, an altered energy and fatty acid metabolism has been identified in pancreatic CSCs by means of omics analyses [42]. Although the malignant dysregulation of translation and metabolic processes in cancer cells compared to noncancer cells is well-established [61], this study uncovers another layer of differences in these pathways and processes between pancreatic CSCs and non-CSCs. Of note, we detected downregulated translation in the TIC subpopulation and, consistently, also reduced mTOR signaling, a major regulator of translation. Whether reduced translation in CSCs is a requirement for the maintenance of a relatively quiescent stemness phenotype similar to normal stem cells remains to be addressed [62].

The PTX and qPCR data revealed strong upregulation of the calcium-binding proteins S100A8 and S100A9 in pancreatic TICs both at the protein and transcript levels. RNA interference-mediated loss of function analysis revealed a crucial contribution of these factors to the tumor-initiating and migratory properties of TICs (Figure 4 and Figure 5). Additionally, tumor growth in a mouse model was significantly reduced in the absence of S100A8 and S100A9, respectively, thereby emphasizing the impact and pathophysiological relevance of our findings (Figure 6). Previous studies have shown that members of the S100 protein family are associated with various cancer entities; both S100A8 and S100A9 can promote proliferation, survival, and metastasis (reviewed in References [63,64]). Moreover, the implications of S100 proteins in pancreatic cancer were extensively reviewed in Reference [65]. However, to our knowledge, we here report, for the first time, the crucial functions of these proteins regarding the tumor-initiating and metastatic properties of TICs.

S100A8 and S100A9 can form heterodimers that also act as a cytokine by augmenting leukocyte recruitment to inflammatory sites (reviewed in Reference [66]). According to their chemotactic functions [67], the most prominent role for S100A8/A9 in malignancies is associated with the extracellular secreted form of S100A8/A9 proteins. Consistent with our data, S100A8/A9 also enhance migration [68,69]. An intriguing aspect of S100 proteins is that tumors induce the expression of S100 proteins by releasing VEGF-A, TGF-β, and TNF-α in metastatic target sites, mostly lung but also liver and kidneys, which serves as a chemo-attractant for metastasizing tumor cells [70,71]. In another study, the family of S100 proteins was found to define invasive properties in pancreatic cancer [72], making them an interesting target for therapy [73]. Due to the ability of S100A8 and S100A9 to form heterodimeric complexes [74] and the fact that some reports observed a loss of one binding partner at the protein level if the other one was knocked-out [75,76], single targeting of either S100A8 and S100A9 might even be sufficient to generate a therapeutic response. However, potential monomeric or homodimeric functions should be considered. Like other S100 proteins, binding and activation to the RAGE receptor by S100A8/A9 and subsequent activation of NF-κB in a positive feedback loop has been discussed as one of their major implications in pancreatic cancer progression [65,77,78]. Of note, our group has previously identified S100A9 to be synergistically regulated by cooperative Hedgehog-EGFR signaling [79], suggesting a possible role in Hedgehog-dependent malignancies including nonmelanoma skin cancer and pancreatic cancer [37,80]. In Supplementary Figure S5, we depict a more comprehensive yet speculative regulatory protein interaction network of S100A8 and S100A9 based on the actual IPA network. Potentially, these S100 proteins may maintain a TIC phenotype by a complex protein network inducing downregulation of p53. How these proteins control the malignant properties of TICs and how plastic these networks are is beyond the scope of this study and will be addressed in the future.

The knockdown of the third candidate protein LGALS3BP efficiently reduced migratory capacities and reduced the number of TI spheres (Figure 4, Figure S7, and Figure 5). However, the influence on the number of TI spheres was not as pronounced as seen for S100 proteins. Like S100A8/A9, LGALS3BP is expressed in a number of human cancer entities, though its putative oncogenic role is only poorly understood. Notably, high LGALS3BP expression is a marker of activated tumor-stroma [81], angiogenesis [82], and metastasis, and high LGALS3BP levels correlate with poor survival in some types of cancers [83]. Especially, its glycosylation via GALNT6 seems to be crucial for secretion and its cell growth-promoting functions [84]. Although LGALS3BP has been studied as a putative prognostic factor in pancreatic cancer, little about its biological functions in conferring cancer stemness properties is known [85]. Future experiments will be required to address in more detail the actual role of this protein in tumor initiation and growth.

In summary, our findings demonstrate the efficiency of the untargeted PTX approach in combination with gene expression analysis and functional assays for the identification of novel TIC genes that may be also valid for CSCs. Our workflow culminated in the nomination of three putative driver genes of stem cell-like TICs in pancreatic ductal adenocarcinoma that showed promising results in various functional analyses. These findings call for further extension of this experimental strategy to other cancer entities for a better understanding of the molecular processes that determine the malignant phenotype of TICs. The identification of crucial determinative proteins will be essential for the specific targeting of highly malignant cancer cell populations by novel therapeutic approaches.

## Figures and Tables

**Table 1:** Primer sequences for qPCR analysis of selected target genes identified by quantitative proteomics (PTX).

Gene	Forward (5’–3’)	Reverse (5’–3’)
*RPLP0*	GGCACCATTGAAATCCTGAGTGATGTG	TTGCGGACACCCTCCAGGAAGC
*S100A8*	TTGACCGAGCTGGAGAAAGCCTTG	GGTCATCCCTGTAGACGGCATGGA
*S100A9*	GGGAATTCAAAGAGCTGGTGCGAA	TCTTCTCGTGGGAGGCCCAGGTTA
*LGALS3BP*	CCTGGGCTGGCTGAAGAGCAACT	GCCCCGCTGGCTGTCAAAGA
*NDRG1*	AATGACTCGTTACCTGCCGCCCATC	CCCCGATCCCCGACTTTTCTACTCA
*CTSD*	GGGCGAGTACATGATCCCCTGTGAG	CTTCCCGGCCTGCGACACCTT
*ALDH3A1*	CATGTTCTCCAGCAACGACAAGGTGA	AGGCAAGAGCGGCGGTGAGAGA
*CD9*	GTTTGGCTGGGGGCGTGGAA	TGCGCCGATGATGTGGAATTTATTGTC
*ARPC3*	TCGACCCTCAGAATGATAAACCCAGCA	CGGGCTCCCTTCACTGTCCAGGT

**Table 2:** Lentiviral shRNA constructs for silencing gene expression via RNA interference.

Target Gene	Sigma Mission shRNA Clone ID
Control	shctrl (shc002 (scrambled non-target shRNA))
*S100A8*	TRCN0000053777
*S100A9*	TRCN0000053803
*LGALS3BP*	TRCN0000029414

**Table 3:** Protein and mRNA abundance changes of selected target genes determined by quantitative PTX and qPCR.

	S100A9	S100A8	LGALS3BP	NDRG1	CTSD	ALDH3A1	CD9	ARPC3
PTX ^a)^	++	++	++	+	+	+	+	+
qPCR ^a)^	+++++	+++++	+++	++++	0	++	+	+
								

^a)^ 3D:2D expression ratios are indicated by 0 (<1.3-fold), + (1.4–2.3-fold), ++ (>2.3–6-fold), +++ (>6–20-fold), ++++ (>20–100-fold), and +++++ (> 100-fold). Genes are ranked in decreasing order according to their differential 3D:2D protein expression levels.

## Supplementary Materials

The following are available online at https://www.mdpi.com/2073-4409/9/6/1397/s1: Figure S1: Tumor growth upon implantation of non-TICs (A) and pancreatic TICs (B); Figure S2: Annotated MS^2^ spectra of unique peptides assigned to S100A8 (A), S100A9 (B), and LGALS3BP (C); Figure S3: Canonical pathways altered in pancreatic TICs; Figure S4: Network connecting significantly altered canonical pathways; Figure S5: Intracellular signaling network obtained by ingenuity pathway analysis; Figure S6: Knockdown efficiency of lentiviral shRNA constructs; Figure S7: Tumor-initiating sphere formation in KRAS-mutated cell line Panc1; Figure S8: Venn diagram comparing significantly regulated proteins identified in this study and those of Dai et al.; SI-Excel: Supplementary excel file containing PTX identification and quantification data.
