# Supplementary material for: Proteins and Molecular Pathways Relevant for the Malignant Properties of Tumor-Initiating Pancreatic Cancer Cells
Source: Cells. 2020 Jun 3;9(6):1397. doi: 10.3390/cells9061397 (PMC7349116; doi:10.3390/cells9061397)
Supplement: Supplementary file 1 [file cells-09-01397-s001.zip › revised_supplementary_material.pdf]

Supplementary Material

Proteins and Molecular Pathways Relevant for the Malignant Properties of Tumor-Initiating Pancreatic Cancer Cells

Lisa Samonig[1a], Andrea Loipetzberger[2a], Constantin Blöchl[1a], Marc Rurik[3], Oliver Kohlbacher[3,4,5,6], Fritz Aberger[2,7*], and Christian G. Huber[1,7*]

[a)]Equally contributing authors

[1] Department of Biosciences, Bioanalytical Research Labs, University of Salzburg, Salzburg, Austria; lisa.samonig@thermofisher.com, constantin.bloechl@sbg.ac.at, c.huber@sbg.ac.at

[2] Department of Biosciences, Molecular Cancer and Stem Cell Research, University of Salzburg, Salzburg, Austria; andrea.loipetzberger@gmail.com; fritz.aberger@sbg.ac.at

[3] Institute for Bioinformatics and Medical Informatics, University of Tübingen, Sand 14, 72076 Tübingen, Germany; rurik@informatik.uni-tuebingen.de; kohlbacher@informatik.uni-tuebingen.de

[4] Biomolecular Interactions, Max Planck Institute for Developmental Biology, Max-Planck-Ring 5, 72076 Tübingen, Germany

[5] Institute for Translational Bioinformatics, University Hospital Tübingen, Hoppe-Seyler-Str. 9, 72076 Tübingen, Germany

[6] Quantitative Biology Center, University of Tübingen, Auf der Morgenstelle 10, 72076 Tübingen, Germany

[7] Department of Biosciences, Cancer Cluster Salzburg, University of Salzburg, Salzburg, Austria

[*]Correspondence: c.huber@sbg.ac.at (C.G.H.), fritz.aberger@sbg.ac.at (F.A.); Tel.: +43-662-8044-5738 (C.G.H), +43-662-8044-5792 (F.A.)

Dr. Christian G. Huber and Dr. Fritz Aberger

Department of Biosciences

University of Salzburg

Hellbrunner Straße 34

5020 Salzburg

Austria

E-mail: c.huber@sbg.ac.at; fritz.aberger@sbg.ac.at

Phone: +43 662 8044 5734; +43 662 8044 5792

Fax: +43 662 8044 5751

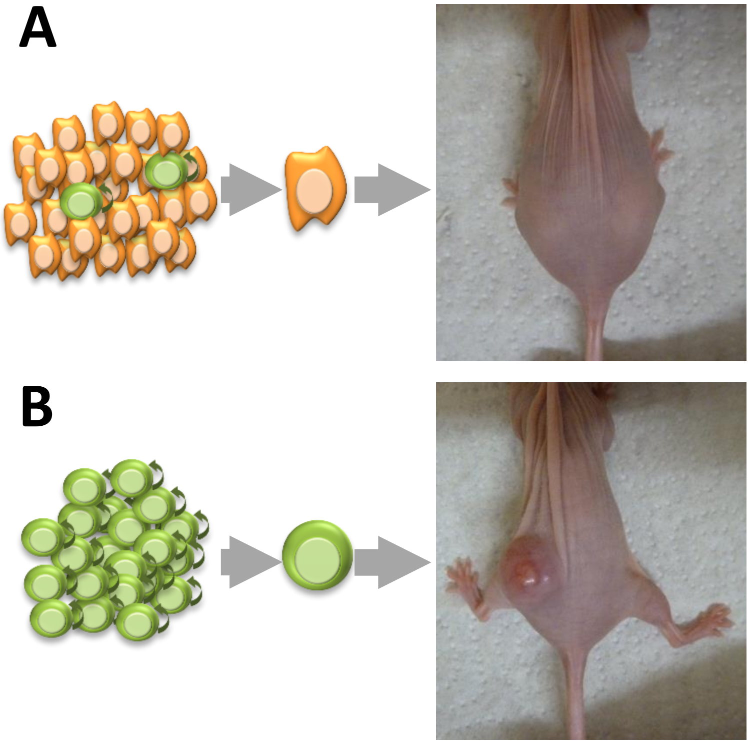

Figure S1. Tumor growth upon implantation of (A) non-TICs and (B) pancreatic TICs. The golden standard assay to validate TICs (green) is the limiting dilution of injected cells to assess in vivo tumor forming capacity. In this assay, 10^2 L3.6sl cells from both the TIC or non-TIC population (orange) were subcutaneously injected into the flanks of nude mice. At this dilution, only the TIC enriched population is able to initiate pancreatic tumor cell growth (n=4/4). No tumor growth was observed for the non-TIC cell population at this dilution (n=0/4). Extensive serial dilution experiments were conducted previously (see Eberl et al., 2012 EMBO Mol Med. 2012 4(3):218-33. Reference [37]).

(A) S100A8

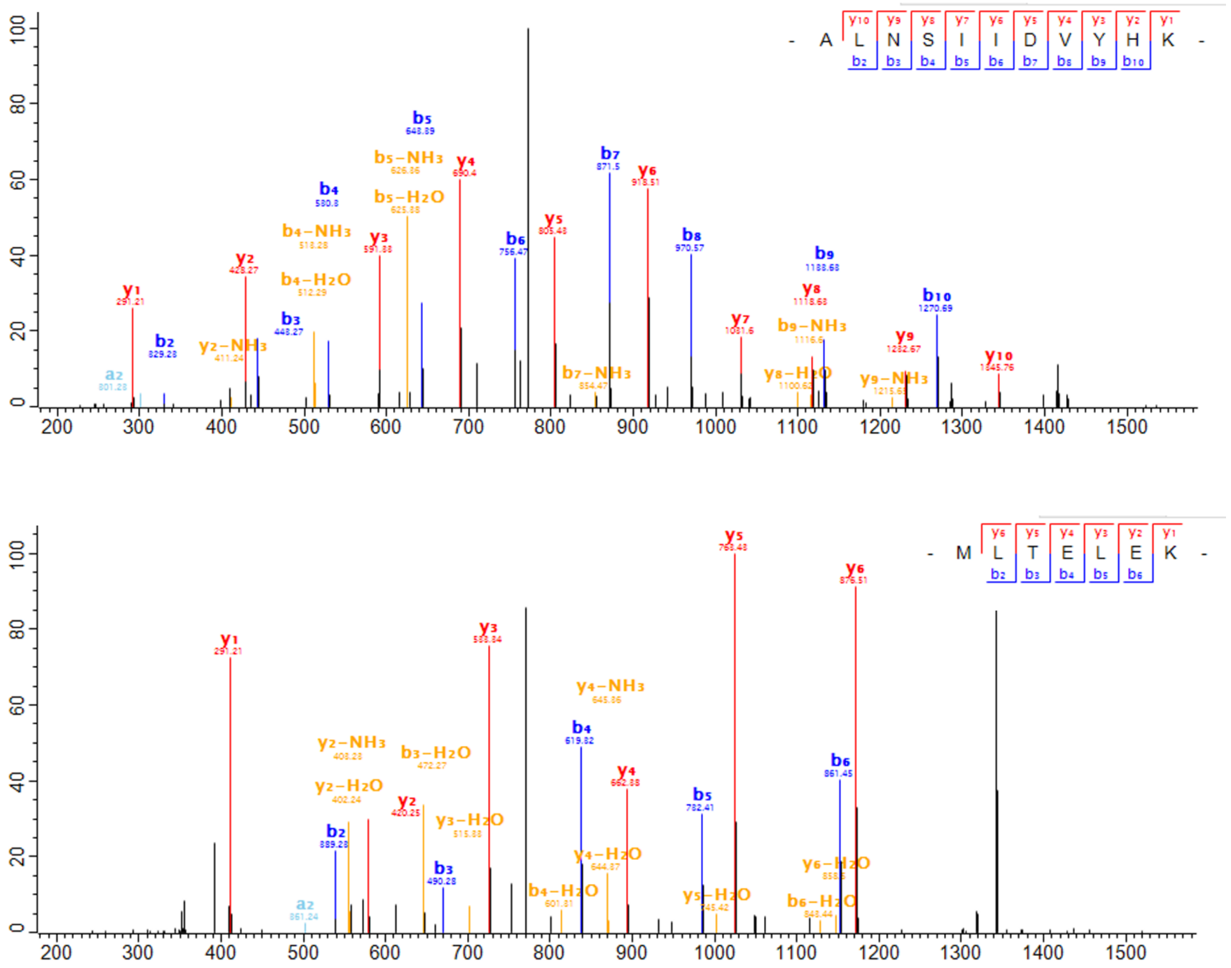

Figure S2A. Annotated MS^2 spectra of two unique peptides assigned to S100A8. Unique peptides were identified after CID and their reporter ions were quantified after HCD. Identified b-ions (blue) and y-ions (red) are matched to the peptide sequence. b-and y-ions showing water or ammonia loss are indicated in yellow, a-ions in light blue.

(B) S100A9

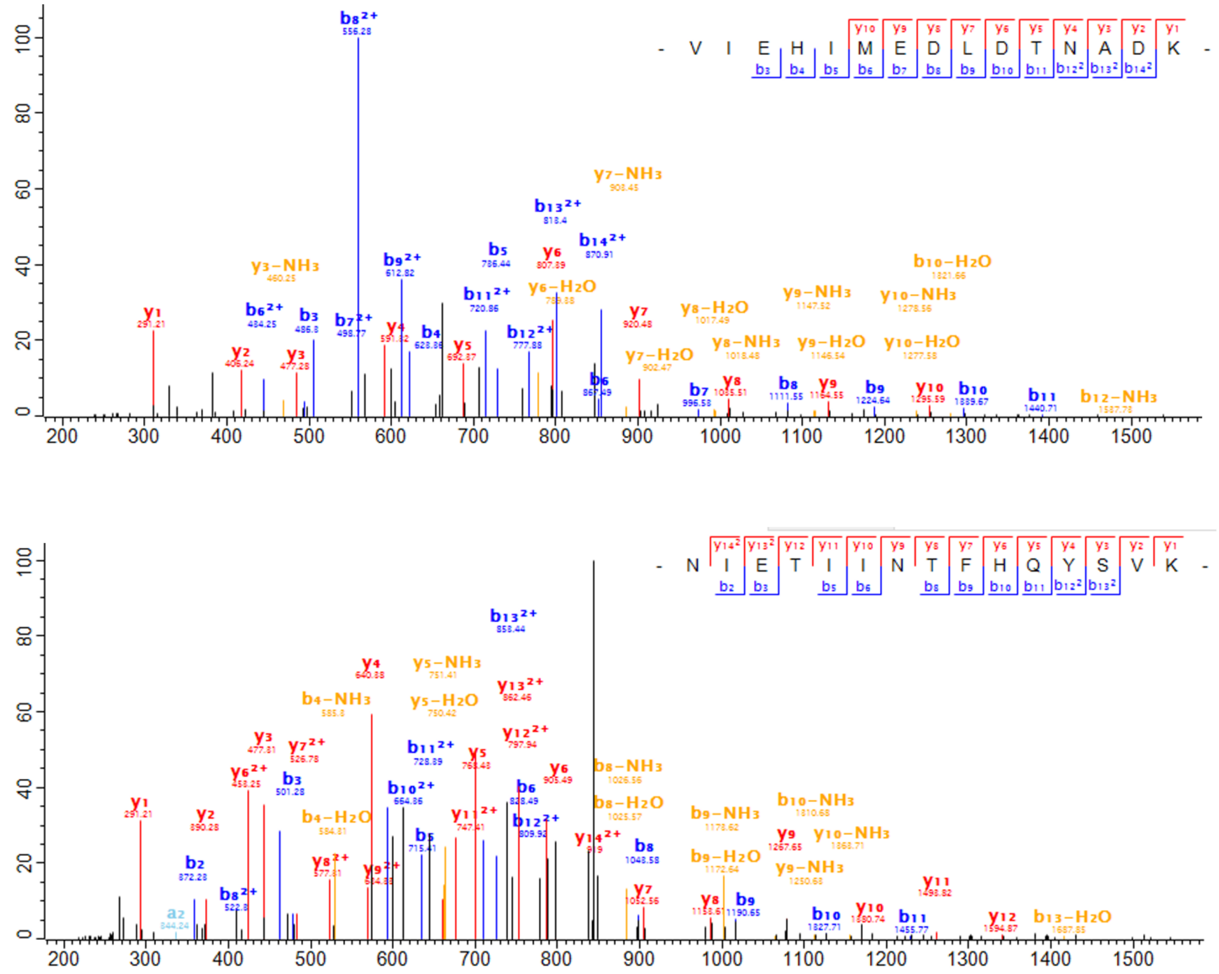

Figure S2B. Annotated MS^2 spectra of two unique peptides assigned to S100A9. Unique peptides were identified after CID and their reporter ions were quantified after HCD. Identified b-ions (blue) and y-ions (red) are matched to the peptide sequence. b-and y-ions showing water or ammonia loss are indicated in yellow, a-ions in light blue.

(C) LGALS3BP

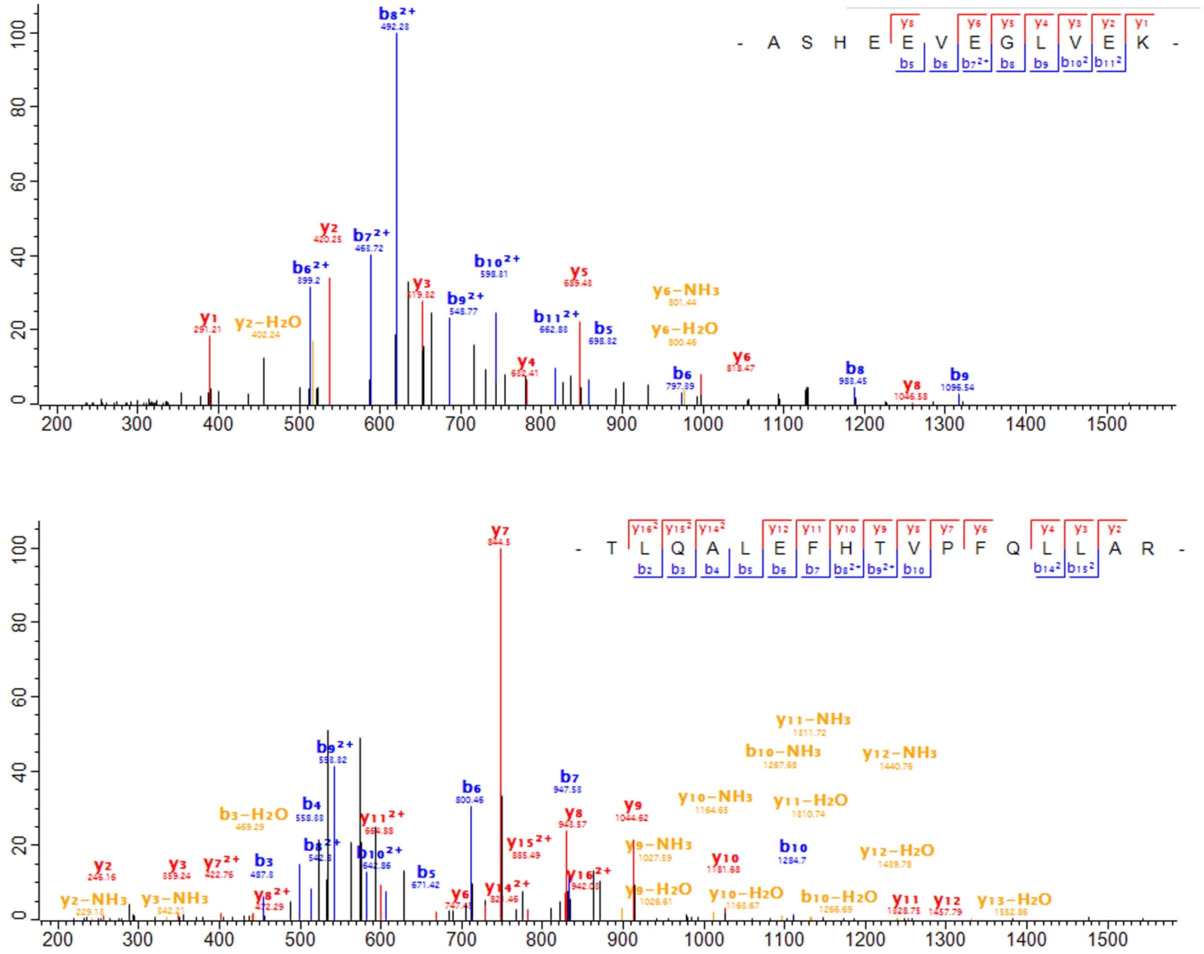

Figure S2C. Annotated MS^2 spectra of two unique peptides assigned to LGALS3BP. Unique peptides were identified after CID and their reporter ions were quantified after HCD. Identified b-ions (blue) and y-ions (red) are matched to the peptide sequence. b-and y-ions showing water or ammonia loss are indicated in yellow, a-ions in light blue.

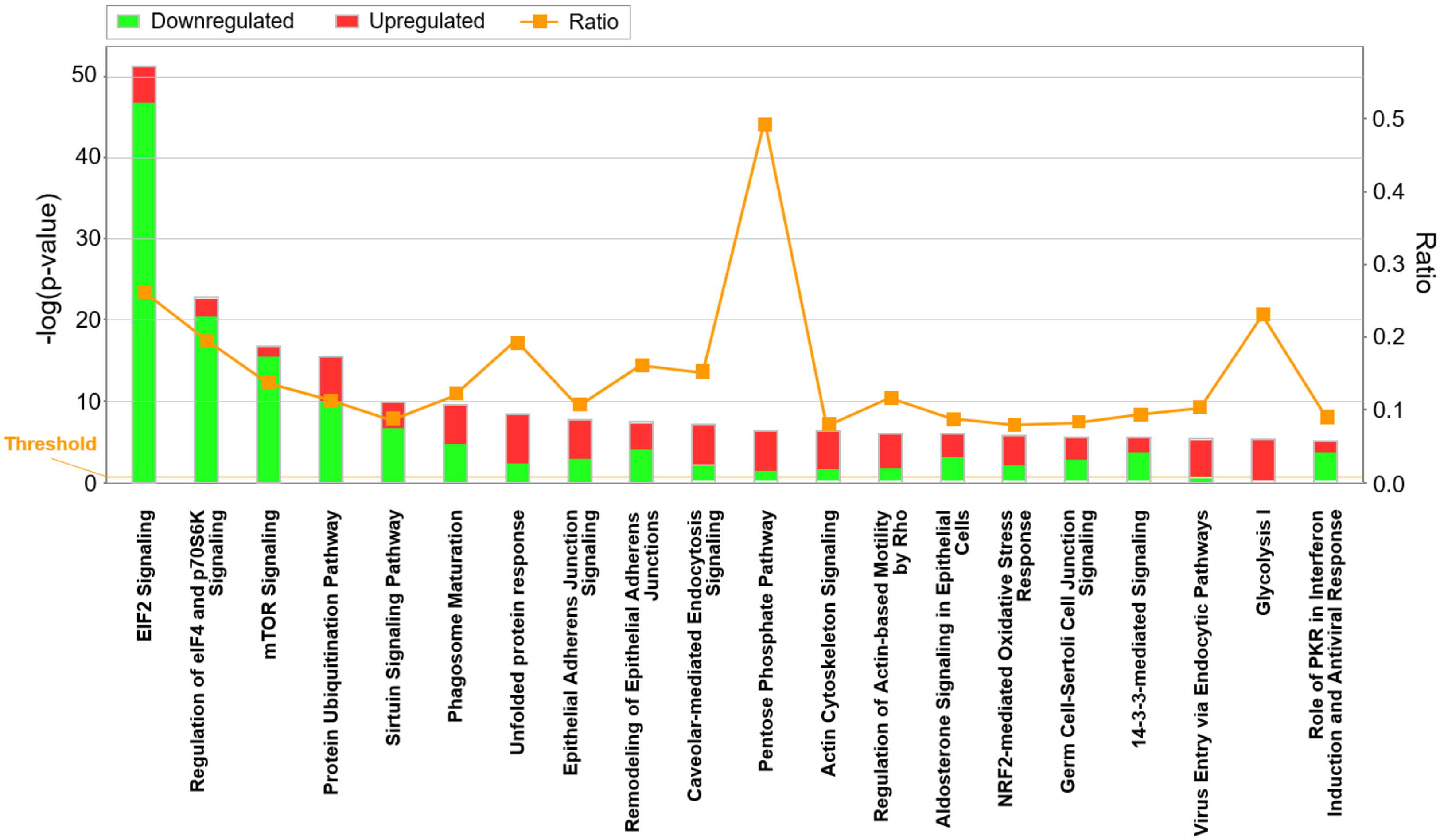

Figure S3. Canonical pathways altered in pancreatic TICs (extension to Figure 3 applying a -log(p-value) cut-off of 5).
IPA pathways altered in pancreatic TICs ranked by significance. For each pathway (x-axis), ratios of significantly up- or downregulated proteins are represented in red and green, respectively. -log(p-value) is the negative decadic logarithm of the probability that the observed association between a specific pathway and the dataset is only due to random chance according to Fisher's exact test; ratio is the quotient between the number of genes detected in our particular analysis (identified *via* the corresponding proteins) and the number of genes contained in the Ingenuity Database for the specific canonical pathway.

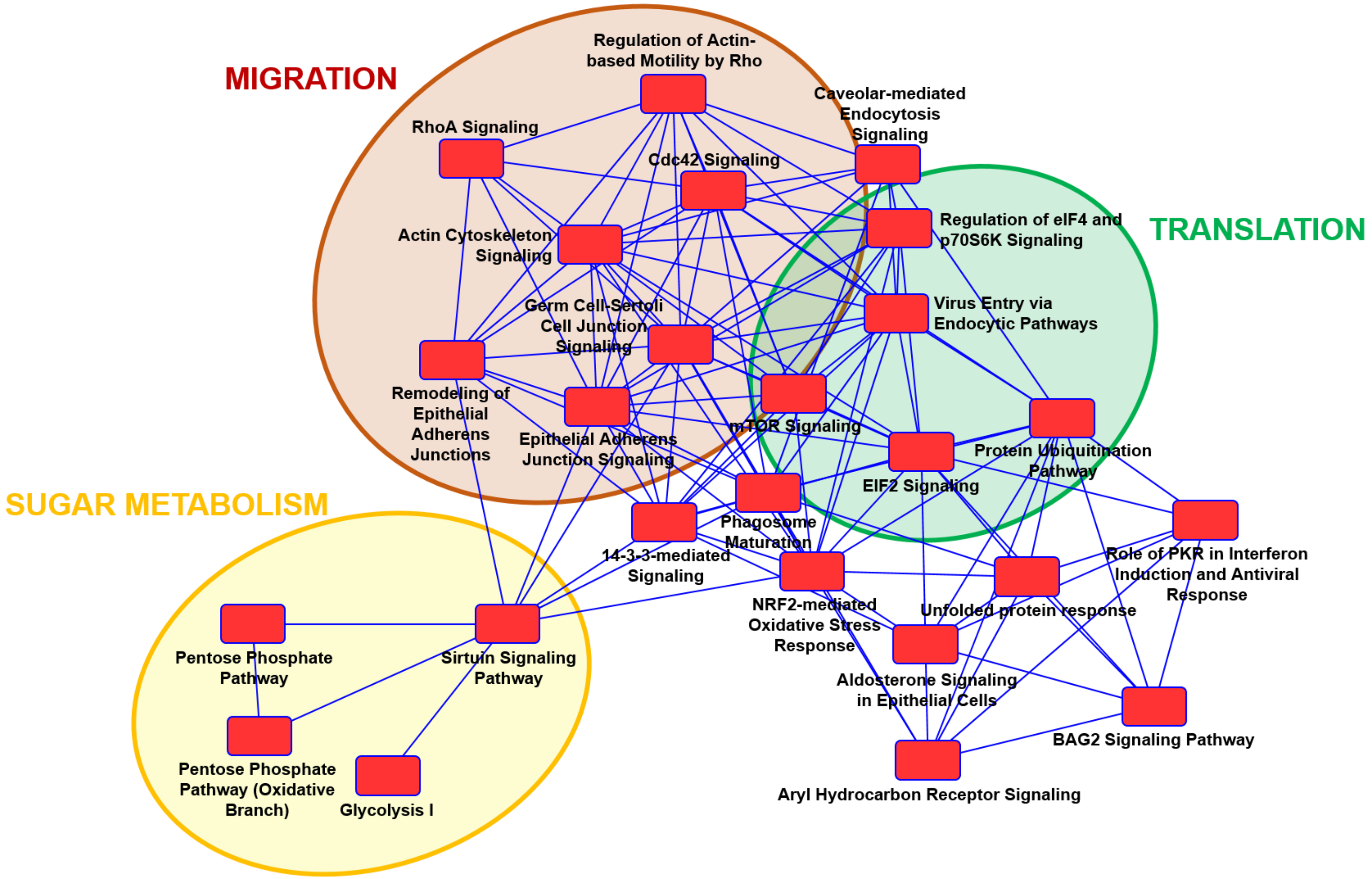

Figure S4. Network connecting significantly altered canonical pathways.

Red boxes depict canonical pathways significantly altered between pancreatic TICs and non-TICs. Blue lines indicate connections of pathways by proteins that were significantly changed in our PTX dataset. The interplay of pathways was visualized by the IPA software. The three major groups, namely sugar metabolism, migration and translation are encircled in yellow, red and green, respectively.

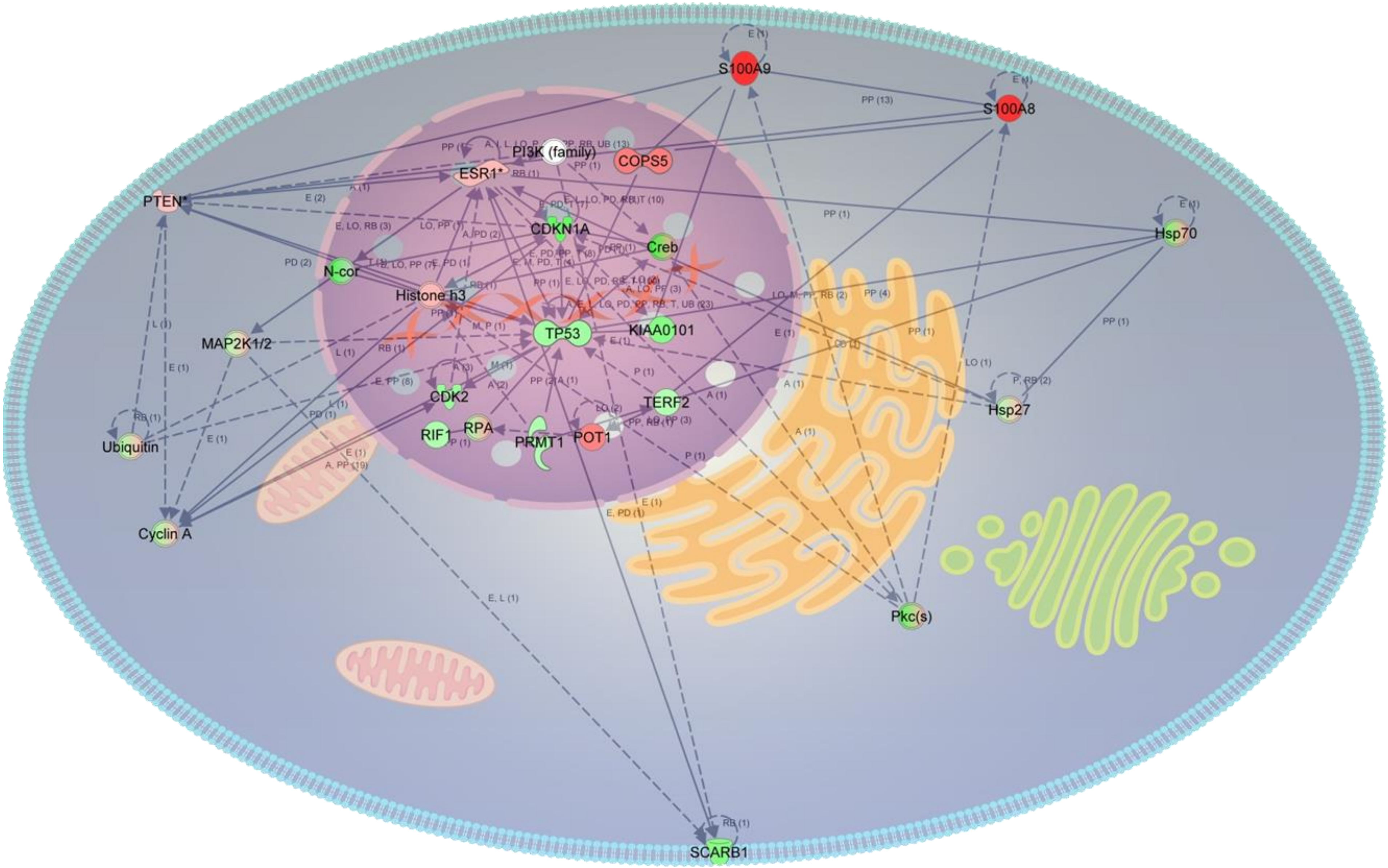

Figure S5. Intracellular signaling network obtained by Ingenuity Pathway Analysis.
The color code indicates regulation found in our analysis: proteins upregulated in cancer stem cells are displayed in red, whereas down-regulated proteins are shown in green, brighter colors indicate a higher degree of regulation.

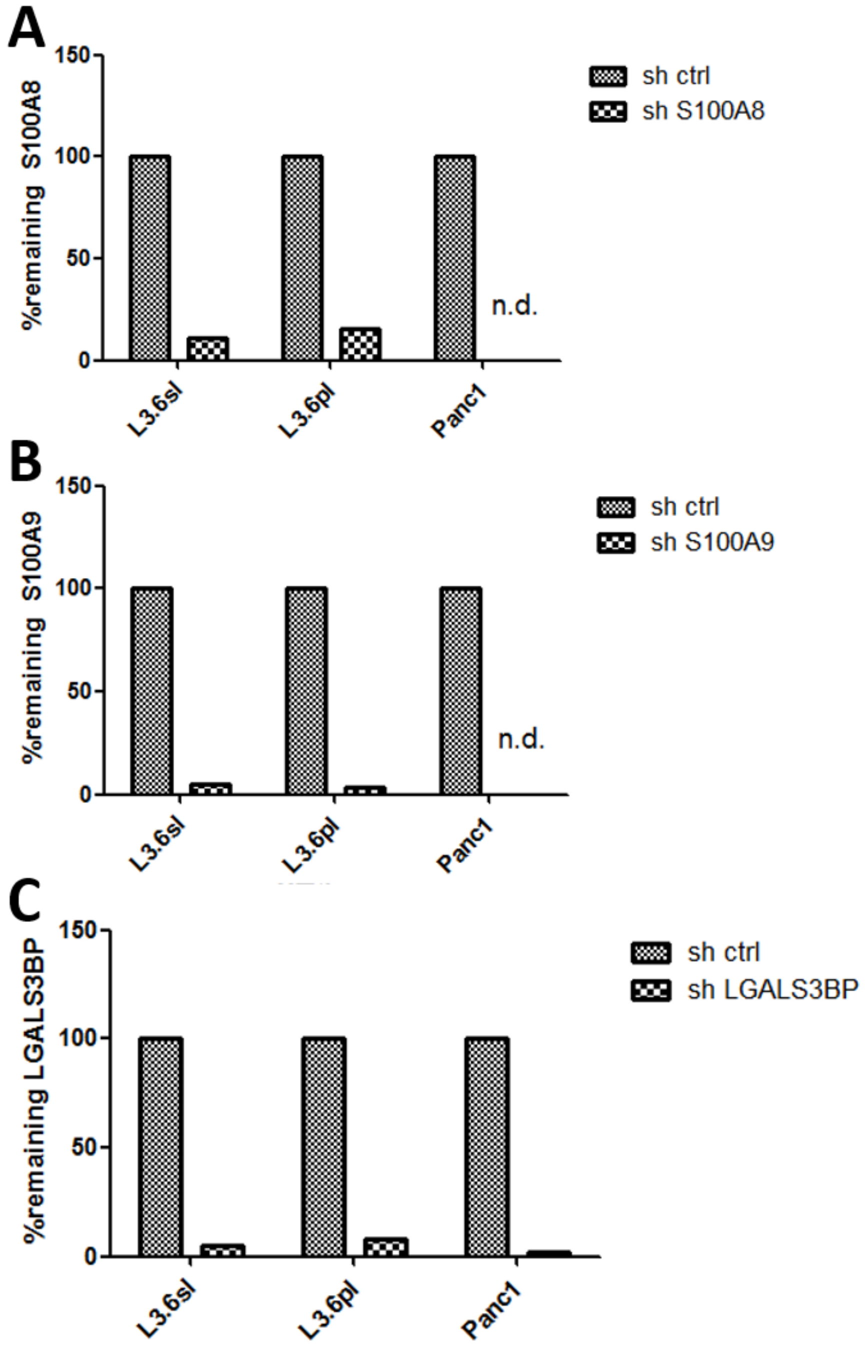

Figure S6. Knockdown efficiency of lentiviral shRNA constructs.

Lentiviral shRNA knockdown efficiency for S100A8 (A), S100A9 (B), and LGAS3BP (C) was assessed by qPCR. Respective mRNA levels in control cells transduced with non-target shRNA (sh ctrl) were used as reference and set to 100 percent.

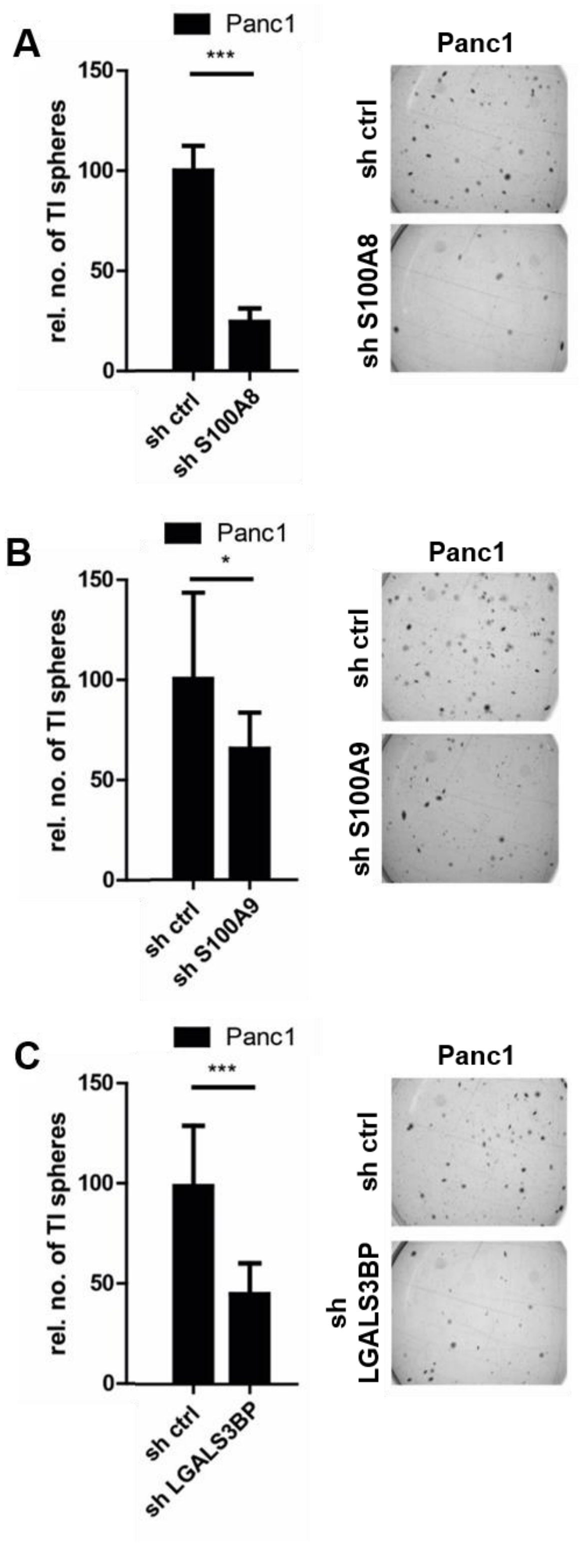

Figure S7. Tumor-initiating (TI) sphere formation in KRAS-mutated cell line Panc1.
Relative number (rel. no.) of spheres upon transduction with control shRNA (sh ctrl) or shRNA against S100A8 (A), S100A9 (B) and LGALS3BP (C) are given as bar charts (left column). Representative images of TI sphere growth are depicted on the right column. Error bars represent standard error of the mean. Statistical significances were assessed by an unpaired t-test (* for $p \leq 0.05$, ** for $p \leq 0.01$ and *** for $p \leq 0.001$, n=12).

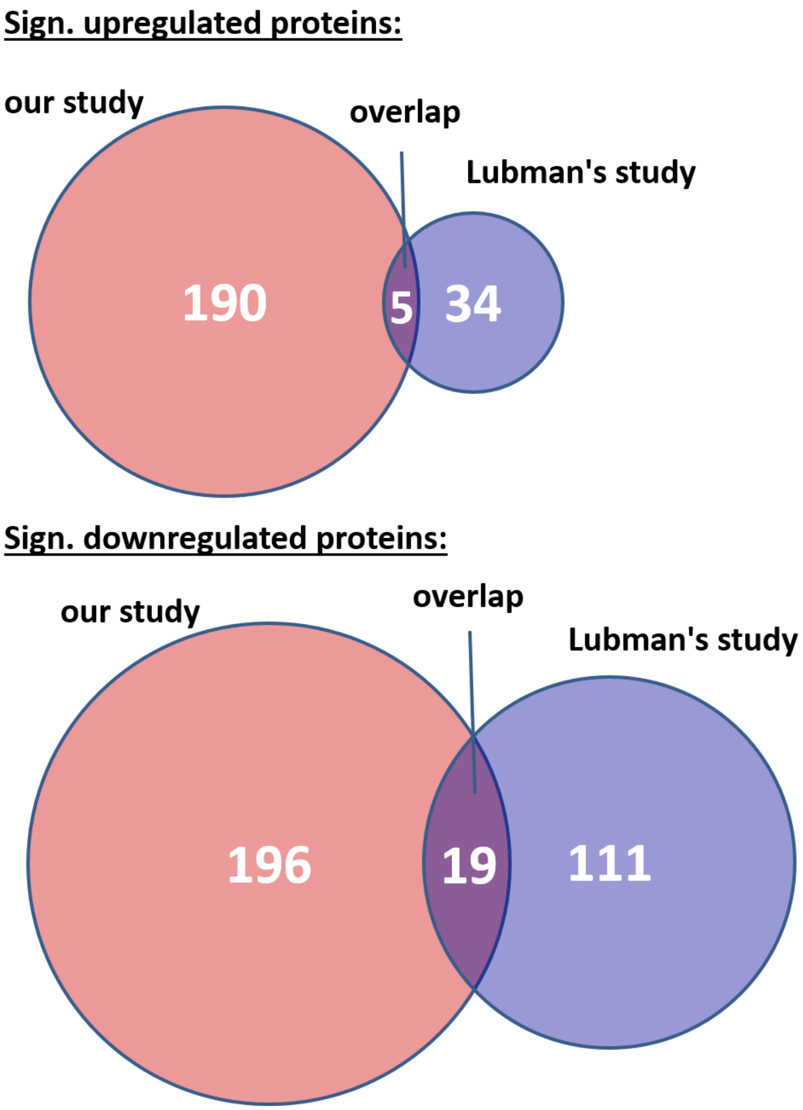

Figure S8. Venn diagram comparing significantly regulated proteins identified in this study and those of Dai et al. (Dai, L., Li, C., Shedden, K. A., Lee, C. J., Quoc, H., Simeone, D. M., and Lubman, D. M. (2010) Quantitative proteomic profiling studies of pancreatic cancer stem cells. *Journal of proteome research* 9, 3394-3402), Reference [41]). Significantly regulated proteins from both studies as well as the overlap are listed in SI-Excel.
